# On Criminal Abortions in America

**Published:** 1860-05

**Authors:** 


*On Criminal Abortions in America.* By Horatio R. Storer, M.D., &c. Philadelphia: J. B. Lippincott & Co. 1860.

This volume is a collection of the essays under the head of "Contributions to Obstetric Jurisprudence," published in the successive issues of the *North American Medico-Chirurgical Review*, for 1859. Our views of the merits of these papers have been previously expressed. In our *Summary* in the September No. of the Monthly, we made reference to them, and endeavored to impress the reader with an idea of their importance. Our over-crowded pages will not permit an analysis of the work, and we must again content ourselves by referring our readers to these contributions of Dr. Storer. The subject of Criminal Abortion is intimately connected with not only the health, but the morals of the community, and cannot be too attentively studied in the light in which Dr. Storer has considered it. O. C. G.

